# Supplementary material for: Identification of photoperiod-induced specific miRNAs in the adrenal glands of Sunite sheep (Ovis aries)
Source: Front Vet Sci. 2022 Jul 22;9:888207. doi: 10.3389/fvets.2022.888207 (PMC9354845; doi:10.3389/fvets.2022.888207)
Supplement: Supplementary file 1 [file Data_Sheet_1.ZIP › Supplementary file/miRDeep2 file.zip!/miRDeep2 file/Novel_Structure/SP42A3/Novel_86.pdf]

Provisional ID	:	NC_019467.2_16142
Score total	:	2.5
Score for star read(s)	:	-1.3
Score for read counts	:	0
Score for mfe	:	2.2
Score for randfold	:	1.6
Score for cons. seed	:	
Total read count	:	75
Mature read count	:	75
Loop read count	:	0
Star read count	:	0

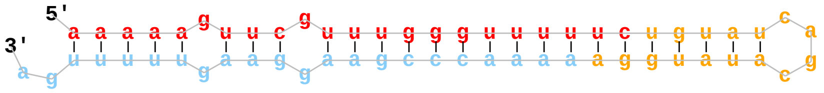

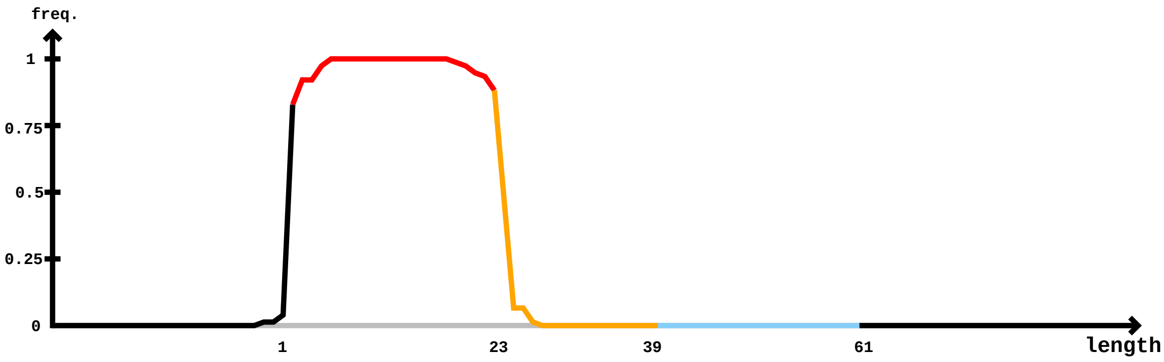

Mature **Star**

```
5'- aggcaucauauauuggguuggcgaaaaaguucguuuggguuuuucuguaucagcauauggaaaaacccgaaggaaguuuuugaacaacccaauaugauauguucaaaaugg   -3'    exp
    .(((((..((((((((((((...(((((.(((.(((((((((((((((((....))))))))))))))))).))).)))))...))))))))))))..))))).......    reads     mm       sample
    ....................gcgaaaaaguucguuuggguu......................................................................    1         0        seq
    ......................Caaaaaguucguuuggguuu.....................................................................    2         1        seq
    .......................aaaaaguucguuugggu.......................................................................    1         0        seq
    .......................aaaaaguucguuuggguuuuC...................................................................    3         1        seq
    .......................aaaaaguucguuuggguuuuuA..................................................................    3         1        seq
    .......................aaaaaguucguuuggguuuuuU..................................................................    8         1        seq
    .......................aaaaaguucguuuggguuuuuc..................................................................    44        0        seq
    .......................aaaaaguuUguuuggguuuuucu.................................................................    1         1        seq
    ........................Gaaaguucguuuggguuuu....................................................................    1         1        seq
    ........................aaaaguucguCuggguuuuu...................................................................    1         1        seq
    ........................aaGaguucguuuggguuuuuc..................................................................    1         1        seq
    ........................aaaaguucguuuggguuuuuA..................................................................    3         1        seq
    ........................aaaaguuGguuuggguuuuucu.................................................................    1         1        seq
    ..........................aaguucguuuggguuuuucu.................................................................    1         0        seq
    ..........................aaguucAuuuggguuuuucugu...............................................................    1         1        seq
    ..........................aaguucguuuggguuuuCcugu...............................................................    2         1        seq
    ...........................aguucguuuggguuuuCcugu...............................................................    1         1        seq
    ...........................aguucguuuggguuuuCcugua..............................................................    1         1        seq
```
